# Pneumatosis Intestinalis With Abdominal Wall Emphysema in Hypothermia

**DOI:** 10.7759/cureus.34909

**Published:** 2023-02-13

**Authors:** Masaatsu Kuwahara, Hiroko Otagaki, Hideaki Imanaka

**Affiliations:** 1 Department of Emergency Medicine, Takarazuka City Hospital, Takarazuka, JPN

**Keywords:** free air, pneumatosis intestinalis, laparotomy decision, wall emphysema, accidental hypothermia

## Abstract

In this report, we present a case in which intestinal and abdominal wall emphysema was observed, but the patient was unconscious due to hypothermia, making it difficult to determine the indication for surgery. Pneumatosis intestinalis (PI) is a pathological condition characterized by the presence of gas within the walls of the small or large intestine and is considered a surgical emergency when accompanied by manifestations of peritonitis on abdominal examination, metabolic acidosis, and lactic acid levels above 2.0 mmol/L. In this specific case, the patient's blood draw results indicated the requirement for an emergency laparotomy; however, the patient's unconscious state became a challenge to make decision on informed consenting. The case illustrates the difficulties encountered in making treatment decisions in critically ill patients and the necessity for thorough assessments and close monitoring of vital signs in such patients.

## Introduction

Pneumatosis intestinalis (PI) is a pathological condition characterized by the presence of gas within the walls of the small or large intestine. The signs of peritonitis on abdominal examination, metabolic acidosis (arterial pH of <7.3 and HCO_3_ of <20 mmol/L), lactic acid of >18 mg/dL, and portal vein gas are considered to necessitate an urgent exploratory laparotomy [1,2]. We present a case in which the results of a blood draw indicated the need for an emergency exploratory laparotomy; however, the patient's inability to confirm the abdominal findings due to unconsciousness, resulting from accidental hypothermia, made it challenging to devise a treatment plan.

## Case presentation

This case report presents the circumstances surrounding an 85-year-old female patient living alone who was taking sodium-glucose cotransporter-2 (SGLT2) inhibitors for preexisting heart failure. The patient's family alerted medical authorities after being unable to reach the patient by phone and subsequently finding her lying on the floor in her home, where the temperature was 3°C and the heating system was turned off. Upon arrival at the hospital, the patient exhibited moderate accidental hypothermia with a bladder temperature of 29.8°C, Glasgow Coma Scale score of 1-1-1, blood pressure of 105/89 mmHg, heart rate of 57 beats per minute, oxygen saturation of 96% on room air, and respiratory rate of 20 breaths per minute. Pupils were 2.5 mm in both right and left eyes, and the light reflex was dull. The abdomen was tender throughout, but the presence of tenderness or peritoneal irritation symptoms was unknown due to impaired consciousness.

Therapeutic interventions were immediately initiated, including warming infusions and surface heating. A blood sample was taken, which revealed a low blood glucose level of 37 mg/dL (normal level: 73-109 mg/dL). Intravenous injection of 50% glucose was administered, resulting in an improvement in blood glucose to 160 mg/dL (normal level: 73-109 mg/dL) after 30 minutes. Despite this improvement, the patient's level of consciousness did not improve. Laboratory analysis revealed no other causes for the patient's impaired consciousness but did reveal high creatinine kinase (CK) levels of 1062 U/L (normal level: 59-248 U/L) and mild acidemia with a pH of 7.293 (normal level: 7.35-7.45), although bicarbonate levels had not decreased to 25.5 mmol/L (normal level: 21-28 mmol/L). Lactic acid levels were slightly elevated at 26 mg/dL (normal level: 4.5-14.4 mg/dL) (Table 1).

**Table 1:** Blood data at admission T-bil, total bilirubin; AST, aspartate aminotransferase; ALT, alanine aminotransferase; UN, urea nitrogen; CRE, creatinine; Na, sodium; K, potassium; CI, chloride ion; CK, creatine kinase; AMY, amylase; CRP, C-reactive protein; GLU, glucose; WBC, white blood cell; Hb, hemoglobin; PLT, platelet; PT, prothrombin time; APTT, activated partial thromboplastin time; Fib, fibrinogen; LAC, lactate

	Blood data	Normal range	Unit
T-bil	1.3	0.4-1.5	mg/dL
AST	58	13-30	U/L
ALT	30	7-23	U/L
UN	38.3	8-20	mg/dL
CRE	0.87	0.46-0.79	mg/dL
Na	138	138-145	mmol
K	4	3.6-4.8	mmol
CI	97	101-108	mmol
CK	1062	41-153	U/L
AMY	86	44-132	U/L
CRP	0.66	<0.14	mg/dL
GLU	36	73-109	mg/dL
WBC	7340	3300-8600	/μl
Hb	12.7	11.6-14.8	g/dL
PLT	26.1×10^4^	15.8-34.8×10^4^	/μl
PT	58	70-130	%
APTT	38.3	<40	
Fib	198	200-400	mg/dL
D-dimer	5.5	<1.0	μg
Lac	26	4.5-14.4	mg/dL

**Figure 1:**
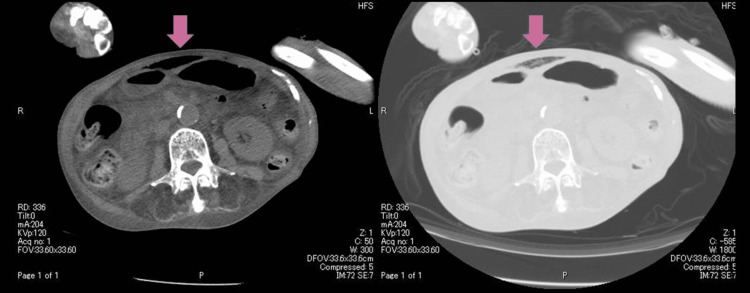
Abdominal wall emphysema on admission In the left image, it is difficult to differentiate between abdominal wall emphysema and intra-abdominal free air. Changing the CT window width as shown in the image on the right reveals that it is abdominal wall emphysema

**Figure 2:**
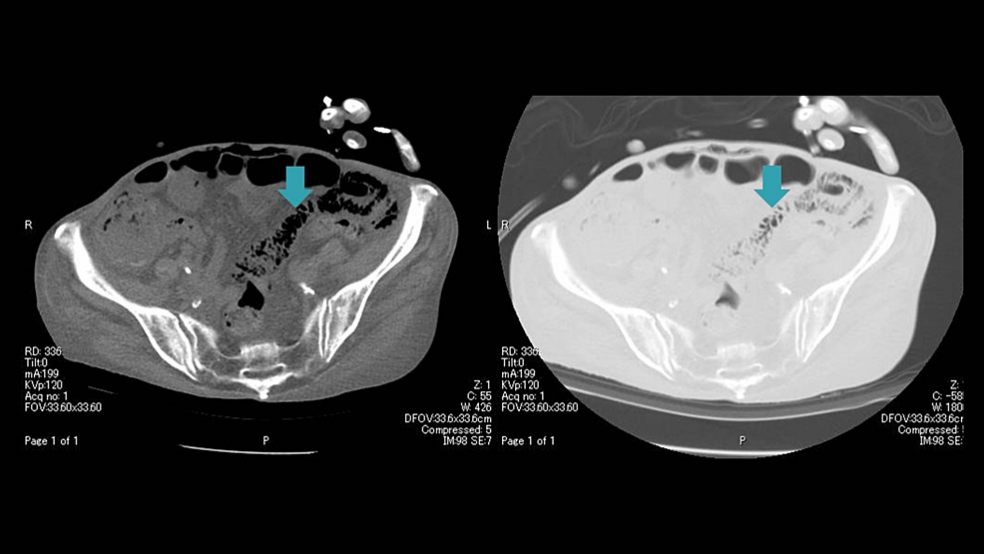
PI on admission PI is seen in the left lower abdomen PI: pneumatosis intestinalis

In an effort to further investigate the cause of the patient's loss of consciousness, a CT scan of the head was performed, which revealed no apparent cause. Because the hypothermia could have been caused by an infection, we performed a thoracoabdominal CT. Additionally, thoracoabdominal CT scans showed PI in the small intestine of the left lower abdomen, as well as suspicious findings consistent with free air (Figures 1-2).

Surgical intervention was considered for possible intestinal perforation, but upon further examination, it was determined that the gas was not intra-abdominal gas but rather extra-abdominal gas, and the patient was diagnosed with abdominal wall emphysema.

Given the patient's unconscious state and inability to accurately examine her abdominal findings, a conservative treatment approach was chosen, including fasting, intravenous infusions, and antimicrobial agents, with close monitoring of the patient's condition and abdominal findings. Fortunately, the patient's condition improved, with creatine kinase (CK) levels decreasing from 1062 U/L on admission to 793 U/L (normal level: 59-248 U/L) the following day, and no deterioration was noted. Additionally, blood culture results were negative for sepsis. Pneumatosis intestinalis had disappeared, and abdominal wall emphysema had decreased on follow-up CT scan. The patient subsequently began oral intake and was transferred to a rehabilitation facility without any worsening of her abdominal findings or general condition.

## Discussion

Hypothermia is characterized by a profound decrease in the core body temperature to less than 35°C. The prevalent categorization of the stages of hypothermia in the literature includes mild hypothermia (core body temperature of 32°C-35°C), moderate hypothermia (28°C-32°C), and severe hypothermia (<28°C) [3,4]. Moderate accidental hypothermia results in a decreased level of consciousness [5]. The patient exhibited moderate hypothermia, the etiology of which was determined to be hypoglycemia caused by the administration of SGLT2 inhibitors for heart failure. We posit that the accidental hypothermia was exacerbated by the inability to activate heating despite a subsequent drop in temperature, further exacerbating the loss of consciousness.

Although sepsis was considered as a differential diagnosis, blood culture results at the time of presentation were negative, and there were no other electrolyte abnormalities or abnormal findings on CT of the head.

A CT scan of the abdomen upon the patient's arrival revealed PI and abdominal wall emphysema, which initially presented a challenge in distinguishing from free air. However, by altering the CT window value, differentiation between free air and PI, abdominal wall emphysema was possible. As reported by Hisanaga et al., this technique of altering the window value is effective in differentiating free air from PI, as was exemplified in the present case [6].

PI is the presence of gas within the walls of the small or large intestine. It is either idiopathic (15%) or secondary (85%) in nature [1]. The etiology of PI can be attributed to mechanical [7], bacterial [8], or biochemical [9] causes, such as alpha-glucosidase internalization.

In the present case, no bacteria were detected in the blood culture, and no alpha-glucosidase was taken internally. Ischemia of the intestine due to circulatory failure caused by hypothermia was also suspected, but trends in CK and lactate (LAC) were also negative for worsening intestinal ischemia.

The management of PI includes emergency exploratory laparotomy in the presence of signs of peritonitis on abdominal examination, metabolic acidosis (arterial pH of <7.3 and HCO_3_ of <20 mmol/L), lactic acid of >18 mg/dL, portal vein gas, and the presence of PI on imaging [2].

In the current case, the patient's arterial pH of 7.293 (normal level: 7.35-7.45) and lactic acid level of 26 mg/dL (normal level: 4.5-14.4 mg/dL) met the criteria for emergency exploratory laparotomy. Initially, the patient's disorientation caused by hypoglycemia and hypothermia precluded an accurate assessment of abdominal findings. After close monitoring of the patient's overall condition, including repeat blood gas tests, a conservative treatment approach was chosen. Once the patient's level of consciousness improved, abdominal findings were confirmed, and there was no indication of peritonitis. No portal vein gas was detected. The difficulty in obtaining abdominal findings in patients with impaired consciousness highlights the importance of obtaining such findings after improvement in consciousness, in order to reduce unnecessary patient invasiveness and to make an accurate determination of the indication for emergency exploratory laparotomy in cases of PI.

## Conclusions

In conclusion, the determination of surgical indication in patients presenting with impaired consciousness can be a challenging task. However, if the disturbance in consciousness is reversible, it is crucial to closely monitor the patient and meticulously evaluate the abdominal findings once the patient regains consciousness to minimize unnecessary patient invasiveness and accurately determine the indication for surgery.
